# The Role of Ion Channel-Related Genes in Autism Spectrum Disorder: A Study Using Next-Generation Sequencing

**DOI:** 10.3389/fgene.2021.595934

**Published:** 2021-10-12

**Authors:** Junghan Lee, Sungji Ha, Jaeun Ahn, Seung-Tae Lee, Jong Rak Choi, Keun-Ah Cheon

**Affiliations:** ^1^ Division of Child and Adolescent Psychiatry, Department of Psychiatry, Severance Hospital, Institute of Behavioral Science in Medicine, Yonsei University College of Medicine, Seoul, South Korea; ^2^ Department of Psychiatry, Institute of Behavioral Science in Medicine, Yonsei University College of Medicine, Seoul, South Korea; ^3^ Department of Laboratory Medicine, Yonsei University College of Medicine, Seoul, South Korea

**Keywords:** autism spectrum disorder, next-generation sequencing, ion channel genes, common variants, restricted repetitive behavior

## Abstract

The clinical heterogeneity of autism spectrum disorder (ASD) is closely associated with the diversity of genes related to ASD pathogenesis. With their low effect size, it has been hard to define the role of common variants of genes in ASD phenotype. In this study, we reviewed genetic results and clinical scores widely used for ASD diagnosis to investigate the role of genes in ASD phenotype considering their functions in molecular pathways. Genetic data from next-generation sequencing (NGS) were collected from 94 participants with ASD. We analyzed enrichment of cellular processes and gene ontology using the Database for Annotation, Visualization, and Integrated Discovery (DAVID). We compared clinical characteristics according to genetic functional characteristics. We found 266 genes containing nonsense, frame shift, missense, and splice site mutations. Results from DAVID revealed significant enrichment for “ion channel” with an enrichment score of 8.84. Moreover, ASD participants carrying mutations in ion channel-related genes showed higher total IQ (*p* = 0.013) and lower repetitive, restricted behavior (RRB)-related scores (*p* = 0.003) and mannerism subscale of social responsiveness scale scores, compared to other participants. Individuals with variants in ion channel genes showed lower RRB scores, suggesting that ion channel genes might be relatively less associated with RRB pathogenesis. These results contribute to understanding of the role of common variants in ASD and could be important in the development of precision medicine of ASD.

## Introduction

Autism spectrum disorder (ASD) is a neurodevelopmental disorder whose essential features include persistent impairment in reciprocal social communication and restricted, repetitive behaviors and interests (American Psychiatric Association and DSM-5 Task Force., 2013). Although ASD is now widely known to the public, with a reported prevalence of 18.5 per 1,000 children aged 8 years in the United States (Maenner et al., 2020), proper diagnosis and treatment are still major challenges for clinicians because of the heterogeneity of the disorder. Regardless of severity, most patients with ASD require suitable therapy considering their individual symptoms (Lord et al., 2018). Among various treatments applied for ASD patients, pharmacotherapy has proven effective in reducing behavioral problems associated with ASD (DeFilippis and Wagner, 2016). Previous studies have reported that over 30% of ASD patients used at least one antipsychotic drug and that the use of medication tends to be higher in ASD children (Rosenberg et al., 2010; Schubart et al., 2014). Pharmacologic treatment in ASD is usually focused on controlling restricted, repetitive behaviors (RRBs), irritability, and aggressive behaviors that are disruptive in behavioral therapy, in social situations, and in daily life (Williamson and Martin, 2012; Fung et al., 2016). Medications are more often prescribed if ASD patients are diagnosed of other comorbid psychiatric illnesses, and antipsychotics are the most frequently prescribed pharmacotherapy in ASD with intellectual disability (Houghton et al., 2017).

The diversity of genes related to ASD pathogenesis appears to be closely associated with the clinical heterogeneity of ASD (Persico and Napolioni, 2013). In addition to ASD-related syndromes or rare chromosomal abnormalities, additive effects from common genetic variants are also known to be related to ASD etiology (Lovato et al., 2019). Moreover, as rare gene variations with a high effect size account only for 10% of idiopathic autism (Persico and Napolioni, 2013), a cumulative effect for common genetic polymorphisms, such as single-nucleotide polymorphism (SNP), with a low effect size are thought to be important in explaining genetic components of ASD (Klei et al., 2012; Gaugler et al., 2014). Meanwhile, recent studies have indicated that common variants could be informative in identifying and diagnosing ASD (Wang and Avillach, 2021) and that cumulative dysfunction of genes by common variants could affect the severity of ASD manifestations (Toma, 2020). Additional research suggests that noncoding variants, as well as single-nucleotide variants and mosaic single-nucleotide variants, are implicated in autism susceptibility (Dias and Walsh, 2020).

However, defining the role of common variants in ASD still faces several obstacles. First, while common variants have been found to be related to ASD etiology in several studies, results have proven difficult to replicate, with an enormous number of genes suspected to be involved in ASD (Lovato et al., 2019). Also, it can be difficult to demonstrate the genetic contribution of a single common gene variant to ASD alone, because ASD shares genetic risks with other psychiatric illnesses, such as schizophrenia, and other neurodevelopmental disorders (Lee et al., 2013). Moreover, stochastic factors during gene expression and environmental factors can also affect the onset of ASD (Geschwind, 2011).

In recent years, next-generation sequencing (NGS), such as whole-genome sequencing, whole-exome sequencing, or clinical exome sequencing, has found use in identifying novel mutations in genes related to ASD (Jiang et al., 2013; Lovato et al., 2019). Most of the genes shown to be associated with ASD can be functionally classified into specific molecular pathways (Sahin and Sur, 2015; Parenti et al., 2020): the pathways include protein synthesis, transcriptional and epigenetic regulation, and synaptic signaling, affecting the functions of neurons and synapses important in neurodevelopment (De Rubeis et al., 2014; Sahin and Sur, 2015). Nevertheless, despite advancements in understanding of the molecular pathology of ASD, it is still unclear how molecular pathway alterations affect ASD phenotypes. For this reason, application of NGS in clinical settings remains limited. Understanding of the linkage between genotypes and ASD phenotypes, however, may help contribute to finally achieving proper diagnosis and predicting prognosis and individualized therapy.

In the present study, we investigated the role of genes in ASD phenotype in consideration of genetic functions in molecular pathways using NGS. We reviewed genetic results and clinical scores clinically used for ASD diagnosis. By excluding rare ASD-related syndromes and rare copy number variants, we only focused on common gene variants. To examine the relationship between ASD phenotype and genotype, we analyzed clinical scores for social function, RRB, and cognitive function in relation to the genetic results.

## Materials and Methods

### Participants

In total, 197 children who underwent NGS for genetic evaluation were included in this study. All children were diagnosed with ASD by specialized child psychiatrists according to the diagnostic criteria suggested in the Diagnostic and Statistical Manual of Mental Disorders (DSM-5) (American Psychiatric Association and DSM-5 Task Force., 2013). The diagnosis of ASD was confirmed with Autism Diagnostic Interview-Revised (Lord et al., 1994) and Autism Diagnostic Observation Schedule-2 scores (Lord et al., 2000). Only children who showed severe autistic symptoms, morphologic problems, or other comorbidities were recommended for genetic evaluation in our clinical setting. Data were collected by retrospectively reviewing medical records for the children. Information on demographics, clinical symptom scores, genetics, comorbidities, and medications was collected. Among 197 children with ASD, we excluded children from analysis if any of the clinical symptom scores described below were missing. Ninety-six children were excluded before analysis due to insufficient clinical data. Afterward, seven children who were diagnosed with ASD-related syndromes (tuberous sclerosis or Rett syndrome) were additionally excluded from this study. This study was approved by the applicable institutional review boards for research with human subjects at Severance Hospital, Yonsei University College of Medicine, where this study was performed. Written informed consent agreeing to donation of human biologic materials was acquired from the participant’s legal guardian/next of kin.

### Clinical Assessments

Autistic characteristics and intellectual function were assessed using the scales and tests listed below. For the assessment of intellectual function, the Korean-Wechsler Intelligence Scale for Children-IV (K-WISC-IV) (Gwak et al., 2011) or the Korean Wechsler Preschool and Primary Scales of Intelligence-IV (K-WPPSI-IV) (Park et al., 2016) was administered depending on the children’s age and ability to perform the test. The Korean-Bayley-III scale was also used for participants who were unable to perform intelligence tests. The Childhood Autism Rating Scale (CARS) was used to distinguish ASD from other developmental disorders and to assess the severity of ASD. A cutoff score of 30 points was applied, and the reliability and validity of the Korean version of CARS have been verified (Shin and Kim, 1998). The Social Communication Questionnaire (SCQ), which is based on the Autism Diagnostic Interview-Revised, was utilized to assess ASD symptoms (Anthony and Catherine, 2003). The Korean version of the SCQ was verified as a reliable and valid screening tool for autism in the Korean population (Kim et al., 2015).

The Social Responsiveness Scale (SRS) is a 65-item questionnaire of social interactions exhibited by children over the past 6 months (Constantino and Gruber, 2012). The test focuses on social impairments in naturalistic social settings and is measured by parents or teachers. It consists of five subscales (social awareness, social cognition, social communication, social motivation, and mannerisms). Each question is scored from 0 to 3 points, and the sum of scores for the social awareness, social cognition, social communication, and social motivation subscales is considered reflective of social communication, a core symptom of ASD in DSM-5. Similarly, the mannerisms subscale represents RRB symptoms, which is also a core symptom of ASD. We previously confirmed the clinical validity of the SRS in Korean children and suggested the relevance of SRS subscales to DSM-5 ASD diagnosis (Cheon et al., 2016). T-scores are used to resolve problems of differences in raw scores by sex or rater (parent or teacher). In this study, we used T-scores of each subscale, as well as total T-scores. T-scores over 75 indicate severe symptoms; T-scores between 60 and 75 are considered indicative of mid-to-moderate severity (Aldridge et al., 2012).

### Next-Generation Sequencing

For exome sequencing, the xGen Inherited Diseases Panel (Integrated DNA Technologies, Coralville, IA, United States) including 4,503 candidate genes was used. The genes included in this panel are known to be related to ASD, intellectual disability, and other neurodevelopmental disorders.

The genomic DNA extracted from the children’s blood was used for library preparation and target capture using a custom panel targeting candidate genes. The NextSeq 550Dx System (Illumina, San Diego, CA, United States) was used to perform massively parallel sequencing. With our custom analysis pipeline, quality control and sequence analysis were proceeded, and copy number analysis was performed (Kim et al., 2019). The GRCh37 (hg19) built as the reference sequence was applied for mapping and variant calling while using the Burrows–Wheeler alignment (BWA) tool (version 0.7.12). HaplotypeCaller and MuTect2 in the GATK package (3.8-0) and VarScan2 (2.4.0) were used to identify single-nucleotide variations (SNVs) and insertion and deletions (indels). Online databases including the Human Gene Mutation Database (HGMD), Online Mendelian Inheritance in Man (OMIM), Clinvar, dbSNP, 1000 Genomes, the Exome Aggregation Consortium (ExAC), the Exome Sequencing Project (ESP), and the Korean Reference Genome Database (KRGDB) were used for analyses and variant annotation.

Classification of variants was conducted using a scoring algorithm implemented in the DxSeq Analyzer (Dxome, Seoul, Korea), based on the standards and guidelines established by the American College of Medical Genetics (ACMG) (Richards et al., 2015). We excluded genetic variants classified as benign or likely benign based on ACMG guidelines in NGS clinical reports by physicians in laboratory medicine. Afterward, variants were lined in order of higher probability of pathogenicity according to ACMG guidelines. Among various variants, we selected five variants with the greatest likelihood of being pathogenetic from each patient.

### Gene Ontology

Using the result of NGS, we analyzed enrichment of cellular processes and gene ontology using the Database for Annotation, Visualization and Integrated Discovery (DAVID) (Huang et al., 2009; Sherman and Lempicki, 2009). Children were then classified according to genetic characteristics.

### Statistical Analysis

Independent t-tests were used to estimate group differences in demographics and clinical scores. The chi-squared test was used for comparing categorical variables. Logistic regression analysis was applied to evaluate the relative risk of a group to another. Statistical significance was defined at *p* < 0.05. All analyses were performed using the Statistical Package for the Social Sciences software (version 25.0; SPSS Inc., Chicago, IL, United States).

## Results

**FIGURE 1:**
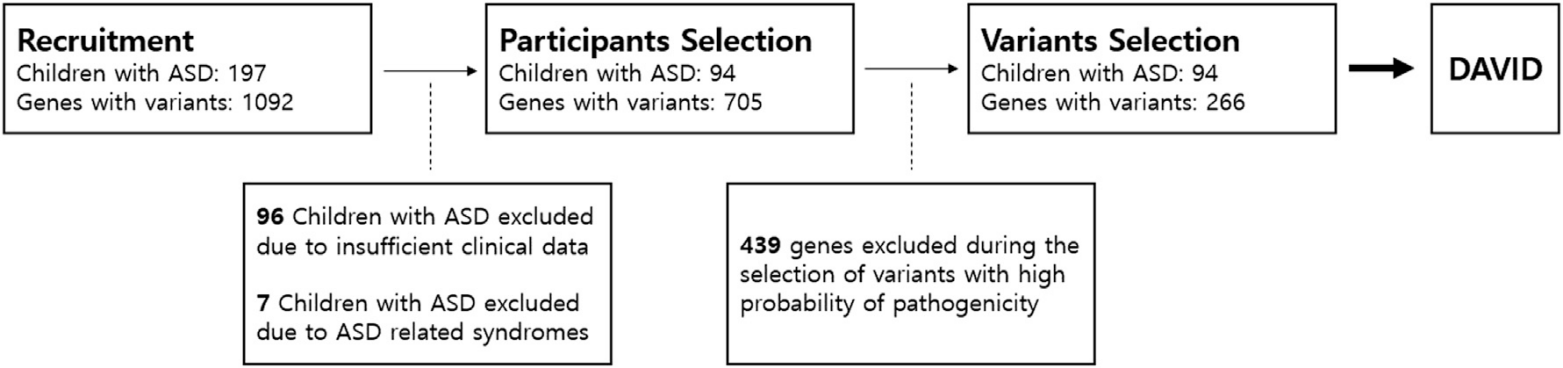
Participants and gene selection. We first recruited 197 children with ASD who underwent next-generation sequencing analysis. In total, 1,092 genes were detected with variants in NGS. We only included participants whose clinical assessment was complete with no missing data. After participant selection, we only selected five variants that were most likely pathogenic in each patient based on ACMG guidelines. A total of 439 genes with variants were excluded. Only 266 genes remained from participant and variant selection. The 266 genes were included in enrichment analysis using the Database for Annotation, Visualization and Integrated Discovery (DAVID). ASD, autism spectrum disorder.

Applying the exclusion criteria, we excluded 94 children with ASD from final analysis (Figure 1). Children with ASD were about 6 years old on average (6.12 years, ranging from 3 years, 6 months to 15 years, 4 months). The male-to-female ratio was about 3:1, and 41 participants were using antipsychotics (aripiprazole or risperidone) because of excessive RRBs or irritability. The average IQ score was 51.87, ranging from 31 to 85. The T-score for total SRS was 85.53 on average, and all subscale T-scores in SRS exceeded 70 on average. CARS scores varied widely, from 21.00 to 51.50 (Table 1).

**TABLE 1:** Demographic and clinical data for children with ASD and group comparisons.

	All (*n* = 94)	Variants in ion channel genes (*n* = 37)	No variants in ion channel genes (*n* = 57)	*p* value
Male:female	63:31	26:11	37:20	
Age (years)	6y + 0.12m	5y + 6.81m	6y + 3.56m	0.141
IQ	51.87	55.22	49.70	0.013*
SRS_T	85.53	81.64	87.98	0.104
SCQ	16.42	15.92	16.76	0.563
CARS	32.09	31.55	32.45	0.404
Medication (*n*)	37	11	26	0.035*

Independent *t* tests were performed to compare average values for age, IQ, SRS_T, SCQ, and CARS between two groups. The chi-square test was proceeded to analyze correlations between ion channel gene variants and medication use. ASD, autism spectrum disorder; IQ, Intelligence Quotient; SRS_T, Social responsiveness scale total score; SCQ, Social Communication Questionnaire; CARS, Childhood Autism Rating Scales. *p < 0.05.

Excluding known benign variants, we found that children carried 0 to 34 variants, with 14.44 variants per child on average. We collected up to five SNVs in children with ASD that had the highest probability of being pathogenic according to ACMG guidelines. In total, we collected 266 genes containing nonsense, frame shift, missense, and splice site mutations. More than one-third of genes (91 genes) overlapped at least twice. Variants in *TSC2* (12 times), *RAI1* (9 times), *CHD7* (7 times), and *RELN* (7 times) were most frequently found among ASD children.

**FIGURE 2:**
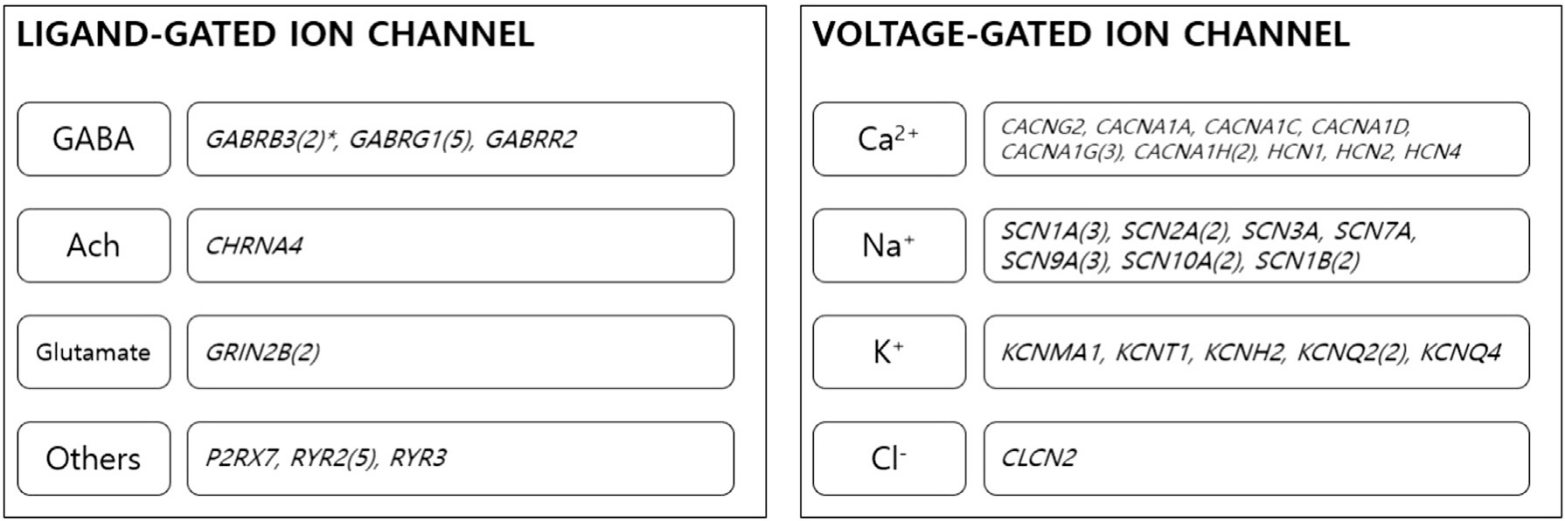
Ion channel genes. Thirty genes were classified as ion channel-related genes. The genes were involved in the function of several ion channels. *Numbers in brackets stand for number of overlapping genes among children with ASD. GABA, gamma aminobutyric acid; Ach, acetylcholine.

Results from DAVID highlighted significant enrichment for “ion channel” (UP_Keywords), with an enrichment score of 8.84 (corrected *p* = 1.9xe-13). In functional annotation clustering, 30 genes were involved in the ion channel cluster: *CACNG2*, *CACNA1A*, *CACNA1C*, *CACNA1D*, *CACNA1G*, and *CACNA1H* were associated with calcium voltage-gated channels; *SCN1A*, *SCN10A*, *SCN2A*, *SCN3A*, *SCN7A*, *SCN9A*, and *SCN1B* were involved in sodium voltage-gated channels; *KCNMA1*, *KCNT1*, *KCNH2*, *KCNQ2*, *KCNQ4*, *HCN1*, *HCN2*, and *HCN4* were related to potassium channels; and *CLCN2* and *CHRNA4* were involved in chloride channel function. *GABRB3*, *GABRG1*, *GABRR2*, *GRIN2B*, *P2RX7*, *RYR2*, and *RYR3* were also highlighted in ion channel functional cluster annotation (Figure 2). Details on variants of ion channel related genes are described in Supplementary Material S1.

**FIGURE 3:**
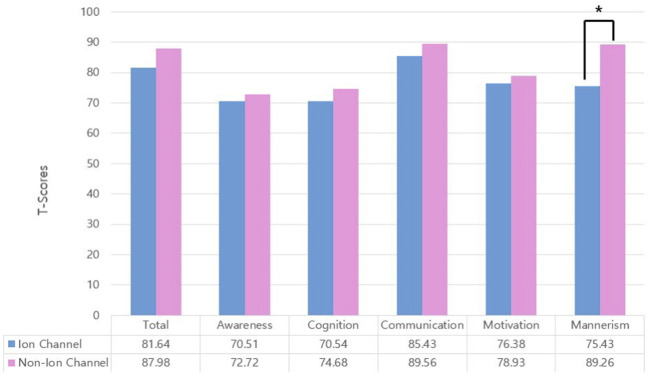
Group comparison of Social Responsiveness Scale (SRS) total T-scores and subscale T-scores. Comparison of children with variants in ion channel-related genes versus others. Only mannerism subscale scores were significantly different (*p = 0.003). **Total:** SRS total T-score; **awareness:** social awareness subscale T-score; **cognition:** social cognition subscale T-score; **communication:** social communication subscale T-score; **motivation:** social motivation subscale T-score; **mannerism:** mannerism subscale T-score.

In our data, 37 children with ASD had at least one variant in a gene involved in ion channel function. ASD children carrying variants in genes related to ion channels (ion channel group) showed significantly higher IQ (*p* = 0.013) and mannerism subscale scores in SRS (*p* = 0.003) than other children that did not. Other clinical scores were not significantly different between groups (Table 1; Figure 3). Chi-square analysis indicated that the ion channel group and medication use were significantly related (*p* = 0.035). On the other hand, children with ASD who were on pharmacotherapy showed significantly lower IQ (*p* = 0.009) and higher CARS scores (*p* < 0.001). There were no differences in SRS total/subscale scores between participants with and without pharmacotherapy (Table 2). Univariate logistic regression analysis revealed that the ion channel group was at a lower risk of undergoing pharmacotherapy, compared to other children (odds ratio = 0.381, *p* = 0.031). Logistic regression also revealed that ASD children with lower IQ scores were more prone to use medication (odds ratio = 1.058, *p* = 0.012) and that higher CARS score were related to medication use (odds ratio = 1.270, *p* < 0.001). Multivariable logistic regression analysis for medication use in ASD children showed that only a high CARS score was predictive of a greater likelihood of receiving pharmacotherapy in ASD (odds ratio = 1.244, *p* = 0.002) (Table 3).

**TABLE 2:** Differences in clinical scores between ASD children with and without antipsychotics use.

	Antipsychotics use (*n* = 41)	No antipsychotics use (*n* = 53)	*p* value
IQ	48.66	54.36	0.009*
SRS_T	88.02	83.56	0.244
SCQ	17.44	15.61	0.202
CARS	34.41	30.22	<0.001*

ASD, autism spectrum disorder; IQ, Intelligence Quotient; SRS_T, Social responsiveness scale total score; SCQ, Social Communication Questionnaire; CARS, Childhood Autism Rating Scales. *p < 0.05.

**TABLE 3:** Logistic regression analysis for antipsychotics use in ASD.

Variables	Antipsychotics use		
	OR	95% CI	*p* value
Univariable logistic regression analysis			
IQ	1.058	1.012–1.105	0.012*
CARS	1.270	1.113–1.450	<0.001*
Ion Channel Group	0.381	0.159–0.914	0.031*
Multivariable logistic regression analysis			
CARS	1.244	1.085–1.426	0.002*

ASD, autism spectrum disorder; OR, odds ratio; CI, confidence interval; IQ, Intellectual Quotient; CARS, Childhood Autism Rating Scales. p values are calculated using analysis of logistic regression. In multivariable logistic regression analysis, IQ, CARS, and ion channel group were adjusted for antipsychotics use. *p < 0.05.

## Discussion

In this study, we examined the role of common genetic variants in ASD phenotype by comparing clinical scores in ASD children with different genetic characteristics. Functional cluster annotation revealed significant enrichment for genes involved in ion channels. ASD children with ion channel-related genetic variants presented with significantly higher IQ and less severe RRBs, leading to less exposure to antipsychotics. Our findings suggested that different molecular pathways regulated by related genes are associated with the different aspect of ASD phenotype. Finding the linkage between the molecular pathway and ASD characteristics may contribute to predict prognosis and precision medicine in ASD at the clinical site.

As NGS was proceeded for comparatively severe ASD patients in clinic, the average of IQ was 51.87, and the average of SRS total T-score was within severe criteria. Considering the result of genetic analysis that children with ASD possess more than 10 variants on average, additive genetic burdens of variants might have played a role in severe symptoms (Pizzo et al., 2019).

Impacting brain development in the prenatal period, defects in ion channels in the brain are critical not only in the pathogenesis of epilepsy but also in other neurodevelopmental disorders, including ASD (D'Adamo et al., 2020; Smith and Walsh, 2020). Mutations in ion channel-related genes seem to induce loss of function or gain of function of cell signaling (Imbrici et al., 2016), resulting in impairment of neuronal networks (Sanders et al., 2018). Although it would be difficult to discriminate the specific function of a particular ion channel in ASD pathogenesis, imbalances in excitation and inhibition have been emphasized in the development of neurodevelopmental disorders (Rubenstein and Merzenich, 2003). Additionally, both excitation and inhibition might play roles in complex neuronal circuits (Nelson and Valakh, 2015). In this study, genes associated with several ion channels were included in a functional cluster. Among 30 genes in the ion channel functional cluster, several genes appeared repeatedly in more than two children with ASD. *GABRG1*, one of the most frequently detected genes in our data, is a gamma-aminobutyric acid (GABA) receptor subunit gene. Although it is not yet clear whether mutated *GABRG1* directly affects ASD pathogenesis, studies have highlighted GABA receptor genes as potentially important in ASD (Ma et al., 2005): for example, GABA receptor density was found to be reduced in ASD (Blatt et al., 2001). Interestingly, a GABA gene cluster on human chromosome 4 was shown to be related to vulnerability to social context in youth (Villafuerte et al., 2014; Trucco et al., 2020), suggesting that these genes affect social functioning. Another frequently detected gene in our data was *RYR2*, which is involved in calcium channel activation in the outer membrane of the endoplasmic reticulum (George et al., 2003). The gene is usually associated with dysregulation of cardiac muscles, but when expressed in the brain, the gene may take part in social functioning and delayed development (Lu and Cantor, 2012). Mutations in calcium voltage-gated channel-related genes (*CACNA1A*, *CACNA1C*, *CACNA1D*, *CACNA1G*, and *CACNA1H*) and sodium voltage-gated channel-related genes (*SCN1A*, *SCN2A*, *SCN3A*, *SCN7A*, *SCN9A*, *SCN10A*, and *SCN1B*) were also detected by our clinical exome sequencing, in line with previous studies reporting genetic associations with ASD (Schmunk and Gargus, 2013). Although it is unclear how much these genes contribute to actual ASD pathogenesis, they do, at the very least, appear to enhance susceptibility to ASD (Schmunk and Gargus, 2013).

One particularly noteworthy finding in this study is that children with variants in ion channel-related genes showed significantly lower RRB scores, suggesting that channelopathy is unlikely to be associated with RRB pathophysiology. In this study, lower RRB scores were significantly related to less use of antipsychotics, an important issue in managing ASD children. Genes related to RRB etiology have been shown to be highly heritable (Ronald et al., 2006) and to be independent of genes affecting social functioning (Ronald et al., 2005). Genetic differences in ASD core symptoms are also supported by differences in RRB symptom severity by sex (Szatmari et al., 2012). Although it remains difficult to explain the genetic differences between social impairment and RRBs, neurobiological factors, such as cortical–basal ganglia pathways, might be closely related (Turner et al., 2006). Considering it is the phenotypic heterogeneity and complex pathophysiology of RRBs in ASD, various genes may be involved (Lewis and Kim, 2009). In addition, neurotransmitter genes, such as dopamine (Lewis and Bodfish, 1998), glutamate (Purcell et al., 2001), serotonin (Di Giovanni et al., 2006), and GABA (Shao et al., 2003) genes, have been shown to be associated with RRBs (Lewis and Kim, 2009). While the cumulative burden of common genetic variants likely affects ASD phenotype the most, we assume that ion channel-related genes may be less connected to RRBs. Although the ion channel group was significantly associated with medication use in our study, the overall severity of ASD represented by CARS scores was more strongly associated with pharmacotherapy of ASD than the RRB score. This might be because RRBs are not the only reason for pharmacotherapy in ASD: emotional problems can also account for antipsychotic use (Stepanova et al., 2017).

There are several limitations to this study. First, the sample size was relatively small, compared to other genetic studies. As this study reviewed medical records retrospectively, we made an effort to include only ASD children with sufficient clinical scores. Also, NGS was only conducted for severe ASD patients at our clinic. As such, the average IQ score was 51.87, and the average total T-score for SRS fell within severe criteria. This limits the generalizability of our results to individuals with less severe ASD. Second, we used both K-WISC-IV and K-WPPSI-IV for intelligence tests because of differences in age at examination, resulting in un-unified subscales of IQ. Third, as reports on genetic results of NGS in Korean individuals with ASD are scarce, we were unable to compare our results within this population. Also, as we only reviewed medical records, we could not compare our results with a healthy control group. Similar studies including healthy controls should be followed to avoid false-positive results. Fourth, considering the preschool age and the low intellectual function, we did not evaluate the comorbidity of attention-deficit/hyperactivity disorder (ADHD) because of the diagnostic instability (Bunte et al., 2014). As ADHD could also possess genetic variants related to the ion channel pathway (Thapar et al., 2016), longitudinal follow-up of comorbidities should be followed. Fifth, we analyzed common variants in genes that seemed to have a low-to-moderate effect size, rather than rare variants. Our selection thereof may put into question if the genetic variants truly affect ASD etiology. Also, considering that genetic analysis has indicated that children with ASD possess more than 10 variants on average, we suspect that additive genetic burden from variants might have played a role in the more severe symptoms seen in our patients (Pizzo et al., 2019). In spite of these weaknesses, we present one possible way in which to interpret the meaning of numerous common variants in ASD. Also, we attempted to discriminate relatively pathogenic variants using ACMG guidelines.

In conclusion, we found several ion channel-related genes to be involved in ASD etiology. Although mutations in ion channel genes are expected to present low-to-moderate effect sizes, they might enhance susceptibility to ASD. Moreover, participants with variants in ion channel genes showed lower RRB scores, suggesting that ion channel genes might not be strongly associated with RRB pathogenesis. These results contribute to helping further understanding of the role of common variants in ASD and could prove to be important in the development of precision medicine for ASD.

## Data Availability

The datasets presented in this article are not readily available because it includes the patient’s genetic data for clinical purpose. Requests to access the datasets should be directed to kacheon@yuhs.ac.
