# Supplementary material for: The Role of Ion Channel-Related Genes in Autism Spectrum Disorder: A Study Using Next-Generation Sequencing
Source: Front Genet. 2021 Oct 12;12:595934. doi: 10.3389/fgene.2021.595934 (PMC8546317; doi:10.3389/fgene.2021.595934)
Supplement: Supplementary file 1 [file Table1.DOCX]

| Gene | Accession | Nucleotide | Amino acid | Minor allele frequency |
| --- | --- | --- | --- | --- |
| GABRG1 | NM_173536.3 | c.143C>T | p.Thr48Met* | 7.51e-5 |
|  |  | c.1106A>T | p.Asp369Val | 4.03e-6 |
|  |  | c.1348G>A | p.Ala450Thr | 2.40e-5 |
|  |  | c.234A>G | p.= |  |
| GABRB3 | NM_001191321.2 | c.5G>A | p.Trp2Ter* | 7.23e-5 |
| GABRR2 | NM_002043.3 | c.80G>A | p.Arg27Gln | 5.98e-5 |
| CHRNA4 | NM_000744.6 | c.1174C>G | p.Pro392Ala |  |
| GRIN2B | NM_000834.3 | c.52G>A | p.Val18Ile | 5.02e-5 |
|  |  | c.609C>T | p.= |  |
| P2RX7 | NM_002562.5 | c.1591G>T | p.Glu531Ter | 2.47e-4 |
| RYR2 | NM_001035.2 | c.8419A>G | p.Ile2807Val | 1.11e-4 |
|  |  | c.8520G>A | p.Met2840Ile |  |
|  |  | c.8522C>A | p.Ala2841Glu |  |
|  |  | c.9876G>C | p.Glu3292Asp |  |
|  |  | c.10708C>T | p.Arg3570Trp | 2.65e-5 |
| RYR3 | NM_001036.3 | c.1566A>T | p.Lys522Asn |  |
| CACNG2 | NM_006078.3 | c.437-2A>G |  |  |
| CACNA1A | NM_001127221.1 | c.2983_2988dup | p.Glu995_Gly996dup |  |
| CACNA1C | NM_000719.6 | c.5729G>A | p.Arg1910Gln |  |
| CACNA1D | NM_000720.3 | c.2242G>A | p.Val748Ile | 4.07e-4 |
| CACNA1G | NM_018896.4 | c.59T>G | p.Met20Arg |  |
|  |  | c.1048-8C>A |  |  |
|  |  | c.7054A>G | p.Met2352Val | 6.44e-5 |
| CACNA1H | NM_021098.2 | c.1943C>T | p.Pro648Leu | 3.19e-5 |
|  |  | c.2350G>A | p.Gly784Ser | 3.22e-5 |
| HCN1 | NM_021072.3 | c.2555C>T | p.Pro852Leu | 3.99e-6 |
| HCN2 | NM_001194.3 | c.2416C>T | p.Pro806Ser | 4.14e-5 |
| HCN4 | NM_005477.2 | c.116G>A | p.Gly39Asp |  |
| SCN1A | NM_006920.4 | c.1893G>T | p.Met631Ile | 3.98e-6 |
|  |  | c.2515G>A | p.Ala839Thr |  |
|  |  | c.2878G>A | p.Val960Ile |  |
| SCN2A | NM_021007.2 | c.982T>G | p.Phe328Val | 5.19e-5 |
|  |  | c.5643G>A | p.= |  |
| SCN3A | NM_006922.3 | c.5873C>G | p.Thr1958Arg | 4.02e-6 |
| SCN7A | NM_002976.3 | c.4136C>T | p.Ala1379Val |  |
| SCN9A | NM_002977.3 | c.29A>G | p.Gln10Arg* | 1.29e-4 |
|  |  | c.688+66G>C |  | 2.45e-4 |
| SCN10A | NM_006514.2 | c.1093-1G>A |  |  |
|  |  | c.4110G>A | p.Met1370Ile | 3.99e-6 |
| SCN1B | NM_001037.4 | c.55G>A | p.Gly19Arg | 1.60e-5 |
|  |  | c.566C>T | p.Thr189Met | 1.88e-4 |
| KCNMA1 | NM_001271520.1 | c.388C>T | p.Arg130Trp |  |
| KCNT1 | NM_020822.2 | c.3157C&gt;T | p.Pro1053Ser |  |
| KCNH2 | NM_000238.3 | c.934C>T | p.Arg312Cys | 9.23e-5 |
| KCNQ2 | NM_172107.3 | c.85C>T | p.Pro29Ser | 2.30e-5 |
|  | NM_172107.2 | c.1343G>A | p.Arg448Gln |  |
| KCNQ4 | NM_004700.3 | c.140T>C | p.Leu47Pro |  |
| CLCN2 | NM_004366.5 | c.2674G>A | p.Asp892Asn | 6.37e-5 |

Supplementary material 1. Ion channel-related genes and its variations selected in the study.
